# Incidental Finding of Venous Air Embolism: A Case Report

**DOI:** 10.7759/cureus.33896

**Published:** 2023-01-17

**Authors:** Sami M Alrasheedi, Lama S Alhumaidan, Abdulmajeed A Alkhathami, Maram Alhati

**Affiliations:** 1 Department of Medicine, Unaizah College of Medicine and Medical Sciences, Qassim University, Unaizah, SAU; 2 College of Medicine, University of Bisha, Bisha, SAU; 3 College of Medicine, Sulaiman AlRajhi University, Al Bukairiyah, SAU

**Keywords:** incidental finding, saudi arabia., case report, transient cerebral ischemia, venous air embolism

## Abstract

Venous air embolism (VAE) is a rare but potentially lethal condition. It has numerous clinical and physiological causes. We present a case report of a 72-year-old Saudi male, known case of diabetes mellitus (DM), hypertension, and ischemic heart disease. The patient came to the emergency room (ER) complaining of the heaviness of the tongue that resolved spontaneously within a few hours. He underwent percutaneous coronary angiography three months ago. The patient with the previously mentioned neurological symptoms, who had been misdiagnosed as having transient cerebral ischemia, was, after a computerized tomography (CT) scan result, diagnosed with venous air embolism. Venous air embolism can occur in situations other than those in which patients are traditionally thought to be at risk, making diagnosis difficult. Any sudden change in mental status and hemodynamic alterations during minimally invasive procedures should raise the physician's suspicion of VAE. Because VAE is an uncommon complication, few cases have been recorded in Saudi Arabia.

## Introduction

Venous air embolism (VAE) is a rare but potentially fatal event. It has many pathological and physiological etiologies. An air embolism occurs when air or gas enters the circulatory system. It can happen iatrogenically through interventional procedures, but it has also been reported as a complication from various conditions ranging from blunt and penetrating trauma to diving and childbirth [1]. The venous air embolism occurs when air enters the systemic venous circulation and reaches the right ventricle.

In contrast, arterial air embolism occurs when air enters the arterial circulation. It is potentially fatal because it can lead to a circulatory deficiency in a body organ with poor collateral circulation [2]. Most gas-related factors, including the patient's general health condition, the body's position, blood solubility, and volume and rate of accumulation, impact how severe the clinical picture is [3-4]. However, many cases of VAE go unreported since they are frequently asymptomatic, and the various testing techniques have differing degrees of sensitivity. As a result, the true incidence of VAE cannot be sufficiently verified [2-3]. VAE is presumably the embolic event that occurs most frequently during surgery [2]. Neurosurgical procedures performed in the sitting position have the highest rate of VAE, with an incidence of approximately 10% for cervical laminectomy and an incidence as high as 80% for seated posterior fossa surgery [4]. At the same time, VAE can occur in 11-97% of patients who undergo obstetric-gynecological operations [5]. According to reports, it can happen in up to 69% of patients undergoing laparoscopic surgery [6]. In orthopedic surgeries, VAE can occur up to 57% of the time [7]. During the invasive monitoring catheter insertion procedure, less than 2% of VAE occurs [8]. It has been demonstrated that VAEs have extraordinarily high fatality rates. In a case series, the outcomes of 119 patients with a VAE were reported in 2010, and those who passed away made up 21% of the total [9]. Additionally, current studies show that a VAE has a fatality rate of 28% [10]. Here we report and discuss the case of a 72-year-old Saudi male who experienced accidental VAE.

## Case presentation

A 72-year-old Saudi male, a known case of ischemic heart disease, diabetes mellitus (DM), and hypertension, underwent percutaneous coronary angiography three months ago. The patient went to the emergency room (ER) with complaints of tongue heaviness (slurred speech) that went away on its own after a few hours, as well as palpitations and abdominal discomfort, but no history of chest pain, vomiting, loose motions, or fever. The next day, the patients started experiencing increased shortness of breath associated with orthopnea, paroxysmal nocturnal dyspnea, sweating, and dizziness.

His physical examination revealed a temperature of 37.1°C, a pulse rate of 84 beats per minute, a blood pressure of 133/73 mmHg, and an oxygen saturation (SpO2) of 96%. The patient looked well - not pale, jaundiced, or cyanosed. He was not in pain or distress. The patient was conscious and oriented to time, place, and person and moving all four limbs with no neurological deficits. He had neck vein engorgement and mild lower limb edema. Upon examination at chest, he had bilateral basal fine crepitation and a normal heart sound. The abdomen was loose and soft on examination. The laboratory tests were requested from ER at the time of presentation and the findings are summarized in Table 1.

**Table 1:** Lab results BUN: blood urea nitrogen, PT: prothrombin time, INR: international normalized ratio, PPT: partial thromboplastin time

parameter	Patient value	Normal value
Sodium concentration	140 MMOL/L	135 - 145 MMOL/L
Creatinine	116 µmol/L	61.9 - 114.9 µmol/L
Calcium	2.2 mmol/L	2.1 – 2.5 mmol/L
Magnesium	0.91 MMOL/L	0.7 – 1.05 MMOL/L
PHOSPHORUS	0.99 MMOL/L	0.87 – 1.45 MMOL/L
Potassium	4.39 MMOL/L	3.5 – 5.1 MMOL/L
Glucose random	8.12 mmol\L	3.9 – 11 mmol\L
Chlorine	107.1 MMOL/L	98 – 107 MMOL/L
BUN	7.1 MMOL/L	2.5 – 6.4 MMOL/L
PT	14.4	9.8 – 12.7
INR	1.14	0.85 – 1.15
PPT	42.9	26.4 - 36
Bilirubin (conjugated)	4.1 UMOL/L	0 – 5 UMOL/L
Alkaline phosphatase	62 U/L	40 – 129 U/L

**Figure 1:**
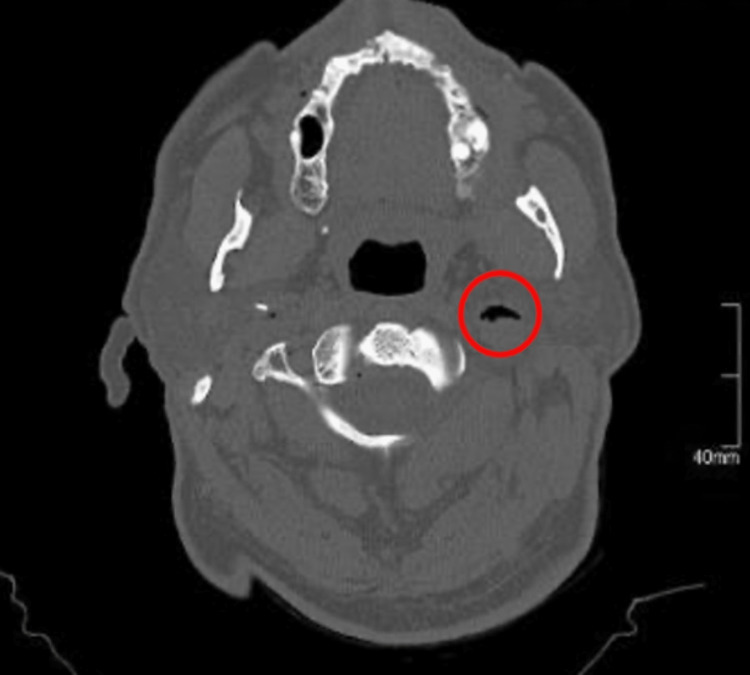
CT imaging of the brain suggests air densities noted within both internal jugular veins, more strongly apparent at the left side.

**Figure 2:**
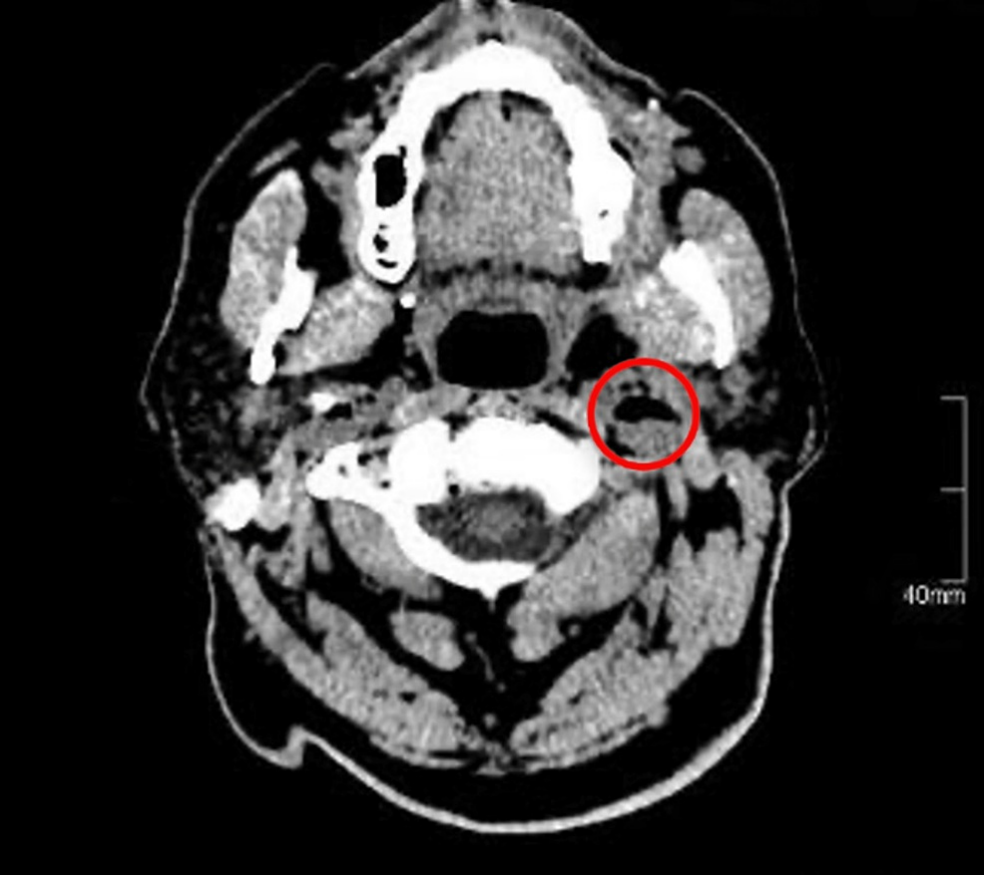
CT imaging of the brain suggests air densities noted within both internal jugular veins, more strongly apparent at the left side.

A chest x-ray was performed at time of admission and showed a congested upper lobe diversion. Brain computed tomography (CT) revealed both internal jugular veins showing evidence of air densities, more so on the left side and to a lesser extent at the cavernous sinuses and right orbit, as well as at the vertebral vessels on both sides (Figures 1, 2).

The right inferior nasal turbinate was hypertrophied, and there was a mild right-sided septal nasal deviation.

Carotid Doppler ultrasound showed bilateral mild atherosclerotic changes with smooth carotid bulb plaques and no significant stenosis. A CT angiography of the head, neck, and chest was not done as the patient has impaired renal function. An echocardiogram was performed and showed impaired left ventricular systolic function, ejection fractions of 35%, multiple wall motion abnormalities, a dilated left ventricle, moderate mitral regurgitation, a mildly calcified aortic valve with no significant gradient, trace tricuspid regurgitation, pulmonary arterial systolic pressure (PASP) of 25-30 mmHg, tricuspid annular plane systolic excursion (TAPSE) of 17 mm, no pericardial effusion, no mass, and no shunt were seen.

The patient was initially misdiagnosed and admitted as a case of transient cerebral ischemic attack, but after finding air in the left jugular vein by accident, we diagnosed it as a case of venous air embolism, along with decompensated heart failure, and coronary artery disease (CAD) post percutaneous coronary intervention (PCI) post-stenting.

The patient was started on injections of enoxaparin (4,000 units) subcutaneously and injections of furosemide (40 mg), and his symptoms then started improving gradually. The patient was discharged with the following medications: insulin, carvedilol, atorvastatin, enoxaparin, aspirin, and furosemide. The patient has to follow up in the following clinics: internal medicine outpatient department (OPD) after one week with renal functions test, DM clinic OPD after two weeks, and cardio OPD after one month.

## Discussion

Even though venous air embolism is rare, it can present at a particular case where one might not reasonably project a lethal complication to occur. There are numerous physiological and pathological causes of it. It could be acquired during diving, after penetrating trauma, mechanical ventilation with high inflation pressure, from introducing a central venous catheter, cardiac catheterization, endoscopic maneuvers, surgical procedures in a sitting position, and sexual intercourse [11]. After it is formed, it may circulate into cerebral venous drainage and occlude it, flow into pulmonary circulation and impede the right heart outflow or the embolus could be shunted through a physiological or pathological shunt (e.g., via patent foramen oval, physiologic intrapulmonary shunts) and move through systemic circulation and occlude any organ vasculature so that the presentation would be variant and dependent on the site of the plug [12].

The incidence of air embolism differs among different studies from 1 in 772 to 2,65 cases per 100.000 hospitalizations [13]. It could be explained by many cases that didn't get a diagnosis as the symptomatology and diagnostic tools are nonspecific, or many cases went asymptotic and unnoticed. While air embolism in cardiac catheterization depends on the operator's skills, the total incidence is almost 0.27%, including unnoticed or unreported asymptomatic air embolisms [14].

The sequelae of air embolism vary from one case to another as it depends on the amount of injected air and its site. It ranges from asymptomatic and without notice to chest pain, hypotension, myocardial ischemia, arrhythmias, and cardiac arrest [15]. Thus, the diagnosis is collectively depending on clinical presentation, history of the presence of risk factors, and use of techniques able to detect air bubbles in the bloodstream, which are precordial Doppler ultrasound probe and transesophageal echocardiography (TEE), and eventually, use of CT to decide the definitive diagnosis and determine the severity of ischemia [3-13].

There is no agreement on specific management for air embolism, so prevention during the procedures that place the patient at risk of air embolism formation is more critical. The system should be well prepared before the procedure; the catheters should be aspirated, connections should be tightened, and the manifold should always be held in an upright position. Most cases of small air embolism are asymptomatic and without hemodynamic complications, which don't need therapy [16]. Severe cases may require more aggressive management composed of endotracheal intonation with 100% oxygen, positioning of the patient in the Trendelenburg and left lateral decubitus to alleviate the amount of air embolism flow to the brain, aspiration of the air bubbles from the right heart via the central venous catheter. Most notably, hyperbaric oxygen therapy could be used in an emergency to reduce the amount of air bubbles [17].

Our patient complained of dizziness, altered speech, and difficulty swallowing, which resolved in a few hours, so we performed a brain CT scan without contrast that showed multiple air density bubbles at the right orbit, cavernous sinuses, the region of the vertebral vessels on both sides, and more obviously at both internal jugular veins. Still, it was more pronounced at the left one. This led the patient to present with the previously mentioned neurological symptoms, which had been misdiagnosed as transient cerebral ischemia and then diagnosed as an air embolism after a CT scan result. The patient has a history of coronary artery disease, hypertension, and diabetes and underwent percutaneous coronary angiography three months before the presentation. It is hypothesized that the procedure was behind the embolism formation, which could have introduced air during the procedure and formed the air embolism iatrogenically. 

## Conclusions

Venous air embolism can occur in people not typically considered at risk, making diagnosis difficult. Any unexpected change in mental status and hemodynamic abnormalities during even minimally invasive operations that are not otherwise explainable and potentially cause contact between air or other gases and the vascular system should raise the suspicion of VAE. Suppose the fear of VAE is confirmed, and there are no contraindications. In that case, it is of primary importance to start heparin, considered a very safe medical procedure if done correctly, as soon as possible to improve the patient's outcome and the correction of the ischemia. As VAE is a rare complication, few cases have been reported in Saudi Arabia. 
